# A Free Community Approach to Classifying Disease

**DOI:** 10.1371/journal.pmed.0010016

**Published:** 2004-11-30

**Authors:** Manuel B Graeber, James Lowe, Bishan Radotra

## Abstract

Defining and classifying disease is at the heart of medical practice, but the process is slow and laborious. A new "open source" approach could be faster and more democratic

Defining and classifying disease is at the heart of medical practice. But the standard approach to classification is slow and laborious. A new approach promises to revolutionise the way in which we classify disease. It involves the free and public sharing of information via the Internet—the so-called open-source, or, perhaps more appropriately termed, “free community,” approach (R. M. Stallman, personal communication).

The International Society for Neuropathology is the first worldwide professional medical organisation to adopt such an approach with its International Classification of Diseases of the Nervous System (ICDNS; see http://www.ICDNS.org). The main characteristics of the ICDNS are free collaboration via the Internet, online access to all collaborative tools via the World Wide Web, global participation, and democratic decision making [1].

## Why We Need a New Approach

Before a disease can be recognised, its nature and the conditions surrounding it must be determined in order to establish criteria for its definition. The more precise a disease definition, the greater the benefit is for the patient, especially where specific treatments are available. Once individual diseases are defined, they can be classified, resulting in the creation of conceptual links that are fundamentally important for medical practice and the advancement of medical knowledge. One example is the conceptual linking of Pick disease, Alzheimer disease, progressive supranuclear palsy, and corticobasal degeneration as members of the group of tauopathies. However, the way in which medical classifications of disease traditionally evolve is problematic.

Usually, small groups of experts meet and decide on a classification that fits best with their personal experience. Classifications then change when new scientific developments are applied to link or separate different conditions. An example is the identification of several pathologically distinct types of frontotemporal dementia based on the application of immunohistochemical staining for tau protein and ubiquitin. The wider medical community subsequently validates these new schemes, provided there is agreement on the basic aspects of any new taxonomic concept. Often, however, consensus only emerges after many years and even decades of controversy and dispute. The World Health Organization's classification of brain tumours [2] is an example of a classification that has taken decades to mature. Thus, the established process is not very effective and is undoubtedly time consuming. It is also occasionally politicised, as “egos” may be unable to resist the temptation of leaving their personal mark, while ignoring the cultural benefit of consensus agreement that results in knowledge that is usable by everyone.

New disease entities are presently emerging at a much higher rate because of advances in biomedicine that were triggered by the Human Genome Project. More and more diseases are being redefined according to molecular criteria. The Lewy body diseases, which share a pathological aggregation of the protein alpha-synuclein (“alpha-synucleinopathies”), are an example of a disease subset now defined by a common molecular pathology. The large field of pathology and the neurosciences are two areas where the translation of morphological phenotypes into molecularly defined entities is already well underway. With the pace of change accelerated by advances in molecular science, we need a much more effective way to develop the debate about medical classifications.

## Open Source and the ICDNS

One effective way to further develop this debate is to adopt the approach used by the free-software and open-source movements [3], which have spawned free software, free operating systems, and free scientific and medical journals. Open source has profoundly important implications for science, technology, and medicine.

The development of global computer networks and the World Wide Web, in particular, have fostered the evolution of free and global sharing of intellectual property. The Open Source Initiative (http://www.opensource.org) is a nonprofit venture dedicated to managing and promoting the open-source idea (Box 1). A related but more radical concept is propagated by the Free (as in freedom) Software Foundation (http://www.fsf.org). The creation of the GNU/Linux operating system (http://www.gnu.org and http://www.linux.org) has resulted from the work of both.

**Box 1. The Open Source Initiative**

“The basic idea behind open source is very simple: When programmers can read, redistribute, and modify the source code for a piece of software, the software evolves. People improve it, people adapt it, people fix bugs. And this can happen at a speed that, if one is used to the slow pace of conventional software development, seems astonishing… Open source software is an idea whose time has finally come. For twenty years it has been building momentum in the technical cultures that built the Internet and the World Wide Web. Now it is breaking out into the commercial world, and that's changing all the rules.” (It should be noted that the Open Source Software Initiative of 1998 was preceded by Richard Stallman's Free Software Movement of 1983 [5].) Source: Open Source Web site (http://www.opensource.org).

The ICDNS was inspired by the free software and open-source approach to software development. The Council of the International Society for Neuropathology approved the ICDNS as a community activity at its last meeting in Turin, Italy, in September of 2003 [4]. The implications of ICDNS go far beyond defining neurological diseases. The benefits include the standardisation of neuropathological training programmes across continents and a new means of direct, professional communication between colleagues from countries all over the globe.

## How ICDNS Works

**Figure 1:**
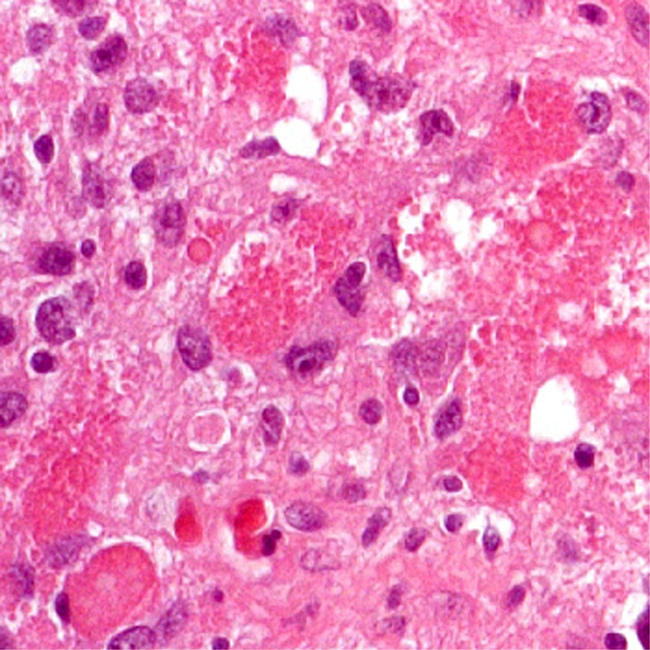
Pilocytic Astrocytoma Future brain tumour classifications will be decided in a democratic way. (Photo: Dr. F. Roncaroli, Department of Neuropathology, Imperial College London)

Diagnostic criteria for all recognised neuropathological diseases are being published on the Web at http://www.ICDNS.org, where the global community of neuropathologists can judge them (Figure 1). No named individual or national group is leading the initiative. Existing classifications are translated into a generic format, avoiding personal as well as institutional names to ensure consistent terminology between related disease processes.

Although still in its early days, definitions for Lewy body disease, Alzheimer disease, and several tumours are now online. Individual ICDNS members (membership is free) as well as expert interest groups propose core definitions, which are then posted so that the global consultation process begins via the World Wide Web. After an online discussion period, ICDNS members holding a specialist certification in diagnostic neuropathology are invited to vote. Comments made online by contributors become part of the history of a disease definition so that nobody is left out and divergent views are not forgotten. The discussion process is thereby open and democratic, allowing wide participation—including from individuals in developing countries who are often excluded in traditional academic discussions.

Different countries around the world show variations in their use of diagnostic criteria and medical classifications, which, in turn, can lead to different treatment approaches. This can certainly create problems when trying to reach a global consensus. However, free access to the information held under the ICDNS open licence may help to minimise these problems by promoting and stimulating collaborative research and the exchange of scientific ideas across the globe.

Certain diseases are far more common in particular parts of the world. In India, for example, neurotuberculosis, cerebral malaria, fungal infections of the central nervous system, human rabies, encephalomyelitis, and cerebrovenous thrombosis are more common than in most developed countries. While the conventional pathology of these diseases is well known—and in some cases we also have expert knowledge on their morphological phenotypes—their exact pathogenesis is not understood, and cellular as well as molecular knowledge is missing. The ICDNS is expected to stimulate local researchers to engage in collaborative international projects in which they can receive feedback via the global consultation process.

Publication of ICDNS criteria occurs under a general public licence. This means that all text can be freely downloaded and republished, avoiding the need for defining basic facts over and over again. Both core definitions and comments may be used immediately for diagnostic purposes as outlined in the guidance section of the ICDNS Web site.

## Future Directions

In the future, definitions of histopathological phenotypes may be linked to clinical as well as molecular biological datasets, such as those obtained from microarrays and combined with imaging parameters. It is obvious that expert consensus on histological phenotypes is required before “genome matching” and similar procedures can be applied to complex diseases in a meaningful way. Most diseases are presently still defined on the basis of their histopathology. Online forums to find diagnostic consensus will provide a very effective means of correlating descriptive data with molecular data. Subsequently, statistical clusters representing “signatures of disease” may be extracted from multidimensional data spaces that will be available online. This opens new roads to link with diagnostic and therapeutic approaches.

It seems reasonable to propose that the adoption of the ICDNS paradigm by other medical specialties would facilitate the development of a comprehensive, global body of medical knowledge. Free access to this knowledge would allow novel, collaborative approaches to be developed to address the most pressing medical problems through supranational concerted efforts. The rather lowly role traditionally ascribed to the exercise of defining and classifying diseases would give way to an appreciation for the key importance of this process as a potentially powerful driver of change.

## Abbreviation

ICDNS: International Classification of Diseases of the Nervous System
